# The Effect of the Mediterranean Diet on Metabolic Health

**DOI:** 10.3390/nu15153397

**Published:** 2023-07-31

**Authors:** Ramona De Amicis, Francesca Menichetti, Alessandro Leone

**Affiliations:** 1 International Center for the Assessment of Nutritional Status and the Development of Dietary Intervention Strategies (ICANS-DIS), Department of Food, Environmental and Nutritional Sciences (DeFENS), University of Milan, 20133 Milan, Italy; ramona.deamicis@unimi.it (R.D.A.); francesca.menichetti@unimi.it (F.M.); 2 IRCCS Istituto Auxologico Italiano, Laboratory of Nutrition and Obesity Research, Department of Endocrine and Metabolic Diseases, 20145 Milan, Italy

The Mediterranean Diet (MedDiet) is one of the healthiest and most balanced dietary patterns worldwide. The MedDiet refers to the traditional eating pattern that became widespread in the early 1950–1960s throughout the Mediterranean. At that time, the MedDiet was considered to be a plant-based dietary pattern, which largely consisted of locally grown food, i.e., fruit and vegetable, nuts, seed, pulses, unprocessed cereals, olive oil as a source of fat and, only in small quantities, dairy products, and meat, or meat products.

This dietary pattern contains a high amount of lipids, particularly olive oil- and nut-derived, healthy unsaturated fats; fiber from pulses, fruits, and vegetables; low levels of non-starchy carbohydrates; and a minimal amount of animal protein that is preferentially obtained from fish and seafood [1].

At the end of 1950s, Ancel Keys and his colleagues were the first to identify the protective effects of the MedDiet against coronary heart disease (CHD) [2]. Since then, the MedDiet has been widely studied, and numerous positive health effects have been reported. Nowadays, the MedDiet has been associated with a better metabolic status [3] and cognitive function [4,5] and a lower risk of major chronic non-communicable diseases, i.e., cardiovascular diseases [6], diabetes [7], cancer [8], metabolic syndrome [9], and mood and eating disorders [10,11,12]. However, the results should be interpreted with caution because of the heterogeneity among these studies, and more high-quality studies are needed to provide robust evidence about the effect of the Mediterranean diet on the incidence of metabolic syndromes and their related comorbidities and the use of pharmacotherapy, as well as to delineate the biological mechanisms responsible for any global health benefits. Therefore, we have organized a Special Issue containing new scientific evidence regarding the effect of the Mediterranean diet on metabolic health. This topic has become even more relevant after the COVID-19 pandemic because of the increased prevalence of obesity [13]. This Special Issue is composed of six articles and two narrative reviews that investigated the relationship between the MedDiet, lifestyles, and metabolic health.

Caldiroli et al. [14] reviewed evidence about the association between the MedDiet and kidney health. This narrative review suggested that the MedDiet could be beneficial both in the prevention and progression of chronic kidney disease, slowing its evolution. Similarly, the MedDiet has been associated with a lower risk of premenstrual syndrome (PMS). Kwon et al. [15] reported that Korean women with a higher adherence to the MedDiet were at lower risk of PMS. On the other hand, the consumption of more bread and snacks is associated with an up to 2.59 times higher risk of PMS. The MedDiet has also been shown to improve the metabolic health of patients with the APOA-5 genetic variant rs662799, which is involved in triglycerides metabolism and linked to metabolic syndrome and CVD. De Luis Roman et al. [16] tested the short (3 months)- and long (9 months)-term effects of the MedDiet on cardiometabolic risk factors among patients with 2 rs662799 genotypes (TT and CT+CC). They observed that the adiposity parameters, systolic pressure, total cholesterol, LDL cholesterol, leptin, adiponectin, and leptin/adiponectin ratio were improved in both genotypes. Moreover, the insulin, HOMA-IR, and triglyceride levels were decreased in non-C allele carriers. The MedDiet seems to also be beneficial for those with chronic inflammatory diseases, including primary Sjögren’s syndrome. Alunno et al. [17] reports that a MedDiet with a low purine content could significantly reduce the serum levels of uric acid (SUA), which, in Sjögren’s syndrome, appears to raise the risk of interstitial lung disease (ILD) and cardiovascular disease (CVD). In a narrative review, Di Majo et al. [18] reviewed the effects of both the Mediterranean and ketogenic diets on people’s immune systems, neuroinflammation, and redox balance. They also developed a ketogenic dietary protocol including low-carbohydrates foods from the Mediterranean diet to reduce the level of neuroinflammation in patients with multiple sclerosis (MS), slowing the progression of the disease. Also Bergia et al. [19] highlighted the importance of considering the glycemic index (GI) in the context of the MedDiet. They conducted a 12-week-long randomized controlled trial among adults at risk of developing diabetes to test the effect of a low-GI or high-GI MedDiet on glucose metabolism. They assessed the postprandial glucose and insulin responses to high- or low-GI meals and the glycemic variability at the baseline and post-intervention. They observed stronger postprandial insulin responses after high-GI meals only at the baseline, but stronger glucose responses both at the baseline and post-intervention. Moreover, despite the average daily glucose concentration decreasing in both groups post-intervention, the indices of 24 h glycemic variability improved only among the adults treated with a low-GI MedDiet. The adherence to the Mediterranean lifestyle (encompassing the MedDiet and other aspects of healthy living, such as food preparation, physical activity, and socializing) has been associated with better metabolic health in patients after a myocardial infarction. Novaković et al. [20] observed that patients who adhere to the Mediterranean lifestyle present with better glucose and lipid profiles both before and after cardiac rehabilitation than the patients who do not adhere to it. Finally, Turki et al. [21] evaluated the changes in diet and lifestyle during the national isolation period among the Tunisian adult population. Despite the proven benefits of the Mediterranean diet, they found that the pandemic affected people’s dietary and lifestyle habits, negatively impacting their adherence to a healthy MedDiet. Thus, sleep quality and physical activity could be additional risk factors for the development of non-communicable diseases.

In summary, the beneficial effects of the MedDiet on metabolic health have been proven. Hence, it would be important to encourage changes in diets that aim to restore the healthy dietary pattern that, in the past, had characterized our diet.
